# Supplementary material for: Deep genome sequencing reveals extensive genetic heterogeneity in early human placentas
Source: Nat Commun. 2025 Aug 23;16:7873. doi: 10.1038/s41467-025-63296-3 (PMC12374992; doi:10.1038/s41467-025-63296-3)

Supplementary Table 1. Sequencing metrics and sex identified from the genomic data for all samples included in the study.

Case No.	Sample type	Duplication rate	Sex	Sequencing depth
1	Chorionic villus sample	13.00%	X0	213.4 x
1	Maternal blood sample	16.10%	XX	48.7 x
1	Paternal blood sample	16.90%	XY	40.3 x
1	Fetal sample (Achilles tendon)	10.90%	X0	144.2 x
1	Placenta 1	10.60%	X0	148.6 x
1	Placenta 2	10.80%	X0	143.7 x
1	Placenta 3	10.40%	X0	161.6 x
1	Placenta 4	11.50%	X0	175.0 x
2	Chorionic villus sample	12.80%	XX	197.7 x
2	Maternal blood sample	11.00%	XX	47.2 x
2	Paternal blood sample	10.70%	XY	42.8 x
2	Fetal sample (Achilles tendon)	11.40%	XX	137.8 x
2	Placenta 1	12.60%	XX	133.4 x
2	Placenta 2	11.90%	XX	102.1 x
2	Placenta 3	12.40%	XX	125.4 x
2	Placenta 4	11.90%	XX	124.4 x
3	Chorionic villus sample	14.80%	XY	188.4 x
3	Maternal blood sample	22.60%	XX	36.2 x
3	Paternal blood sample	22.00%	XY	41.4 x
3	Fetal sample (Achilles tendon)	11.70%	XY	159.3 x
3	Placenta 1	10.40%	XY	118.6 x
3	Placenta 2	11.80%	XY	157.2 x
3	Placenta 3	11.30%	XY	158.6 x
3	Placenta 4	11.80%	XX	153.6 x
4	Amniotic fluid	12.90%	XX	213.6 x
4	Maternal blood sample	7.60%	XX	55.8 x
4	Paternal blood sample	7.90%	XY	56.2 x
4	Fetal sample (Achilles tendon)	10.60%	XX	178.4 x
4	Placenta 1	11.50%	XX	129.3 x
4	Placenta 2	10.90%	XX	103.3 x
4	Placenta 3	11.90%	XX	142.1 x
4	Placenta 4	10.20%	XX	157.5 x
5	Amniotic fluid	12.80%	XX	197.7 x
5	Maternal blood sample	11.00%	XX	47.2 x
5	Paternal blood sample	10.70%	XY	42.8 x
5	Fetal sample (Achilles tendon)	11.40%	XX	137.8 x
5	Placenta 1	12.60%	XX	133.4 x
5	Placenta 2	11.90%	XX	102.1 x
5	Placenta 3	12.40%	XX	125.4 x
5	Placenta 4	11.90%	XX	124.4 x
6	Chorionic villus sample	11.70%	XX	99.3 x
6	Amniotic fluid	13.40%	XX	193.0 x
6	Placenta 1	12.20%	XX	124.3 x
6	Placenta 2	12.40%	XX	150.5 x
6	Placenta 3	12.10%	XX	108.4 x
6	Placenta 4	12.10%	XX	108.0 x
6	Fetal sample (umbilical cord)	12.50%	XX	130.7 x

Supplementary Table 2. Small sequence variants across the coding region in all cases and the identification of them in cell-free DNA analysis.
no - not detected in our analysis of respective sample; Fetal de novo - a cluster of true heterozygous fetal de novo variants.

Case 1

Chromosomal location	Clone	Sample	VAF	Gene	HGVSc	HGVSp	cfDNA analysis	Fetal Fraction
6_142761555_C_T	0	P4	10.96 %	HIVEP2	NM_006734.4:c.5529G>A	NP_006725.3:p.Thr1843=	no	
10_93408905_CGGACG_-	0	P4	8.03 %	MYOF	NM_013451.4:c.606_611delCGTCCG	NP_038479.1:p.Arg204_Val205del	no	
5_131671901_G_C	4	P3	22.1 %	FNIP1	NM_133372.3:c.2543C>G	NP_588613.3:p.Thr848Ser	no	
2_1922818_C_G	5	CVS	5.95 %	MYT1L	NM_015025.4:c.951G>C	NP_055840.2:p.Glu317Asp	VAF 0.3% (2 reads)	
3_184381964_G_T	5	CVS	6.9 %	CHRD	NM_003741.4:c.643G>T	NP_003732.2:p.Asp215Tyr	no	5.29 %
8_25302372_C_T	5	CVS	8.3 %	DOCK5	NM_024940.8:c.894C>T	NP_079216.4:p.Cys298=	no	
22_28795375_C_T	5	CVS	6.34 %	XBP1	NM_001079539.2:c.931G>A	NP_001073007.1:p.Val311Ile	VAF 0.3% (2 reads)	
15_94367702_C_T	Fetal de novo	All samples	See below	MCTP2	NM_018349.4:c.1399C>T	NP_060819.3:p.Leu467Phe	VAF 2.2% (11 reads)	

VAF across samples	CVS	Fetal biopsy	P1	P2	P3	P4
15_94367702_C_T	45.79%	50.00%	43.30%	45.79%	50.70%	47.60%

Case 2

Chromosomal location	Clone	Sample	VAF	Gene	HGVSc	HGVSp	cfDNA analysis	Fetal Fraction
13_33111841_G_C	3	P4	5.52 %	STARD13	NM_178006.4:c.2544C>G	NP_821074.1:p.Asn848Lys	no	
1_241594522_A_C	7	P2	8.43 %	KMO,OPN3	NM_003679.5:c.*2369A>C,NM_014322.3:c.1115T>G	,NP_055137.2:p.Ile372Ser	no	
4_182736946_C_A	7	P2	11.18 %	TENM3	NM_001080477.4:c.3106C>A	NP_001073946.1:p.Leu1036Ile	no	4.36 %
3_44287817_C_A	8	P1	5.67 %	TOPAZ1	NM_001145030.2:c.3659C>A	NP_001138502.1:p.Ala1220Asp	no	
15_81002834_C_T	Fetal de novo	All samples	See below	TLNRD1	NM_022566.3:c.563C>T	NP_072088.1:p.Ser188Leu	VAF 1.6%(10 reads)	
15_96337419_TACGT_-	Fetal de novo	All samples	See below	NR2F2	NM_021005.4:c.1044_1048delCGTTA	NP_066285.1:p.Tyr348Ter	VAF 0.5% (3 reads)	

VAF across samples	CVS	Fetal biopsy	P1	P2	P3	P4
15_81002834_C_T	51.32%	46.76%	46.24%	47.51%	55.32%	45.70%
15_96337419_TACGT_-	46.98%	40.88%	48.00%	56.60%	48.80%	46.36%

Case 3

Chromosomal location	Clone	Sample	VAF	Gene	HGVSc	HGVSp	cfDNA analysis	Fetal Fraction
4_99307854_C_T	1	P3	16.29 %	ADH1B	NM_000668.6:c.1114G>A	NP_000659.2:p.Val372Ile	0.3% VAF (2 reads)	
8_2002852_C_T	1	P3	12.12 %	KBTBD11	NM_014867.3:c.1660C>T	NP_055682.1:p.Arg554Cys	no	
11_55368887_G_T	2	CVS/P4	18.34 % / 9.26 %	OR4A15	NM_001005275.2:c.914G>T	NP_001005275.2:p.Ser305Ile	(VAF 0.2% (only 1 read)	8.60 %
15_40272385_C_T	1	P3	15 %	BUB1B-PAK6,PAK6	NM_001128628.3:c.1020C>T,NM_001395430.1:c.1020C>T	NP_001122100.1:p.Gly340=,NP_001382359.1:p.Gly340=	no	
19_47021847_G_A	1	P3	15.27 %	NPAS1	NM_002517.4:c.358G>A	NP_002508.2:p.Ala120Thr	VAF 0.4% (2 reads)	
22_17181531_G_T	Fetal de novo	All samples	See below	ADA2	NM_001282225.2:c.1488C>A	NP_001269154.1:p.Ile496=	VAF 6.0% (48 reads)	

VAF across samples	CVS	Fetal biopsy	P1	P2	P3	P4
22_17181531_G_T	46.79%	52.46%	53.28%	54.76%	55.63%	17.24%

Case 4

Chromosomal location	Clone	Sample	VAF	Gene	HGVSc	HGVSp	cfDNA analysis	Fetal Fraction
12_78119541_C_A	1	P2	7.46 %	NAV3	NM_001024383.2:c.3345C>A	NP_001019554.1:p.Asp1115Glu	no	
12_29767905_G_A	4	P4	5.31 %	TMTC1	NM_001193451.2:c.473C>T	NP_001180380.1:p.Thr158Ile	no	
2_234069991_G_T	6	P1	6.04 %	SPP2	NM_006944.3:c.614G>T	NP_008875.1:p.Arg205Ile	no	6.95 %
5_61535604_-_C	9	Fetus/AF	4.25 % / 6.01 %	ZSWIM6	NM_020928.2:c.2366dupC	NP_065979.1:p.Leu790Thrfs*42	no - confined fetal mosaicism	
4_182688238_C_T	Fetal de novo	All samples	See below	TENM3	NM_001080477.4:c.2108C>T	NP_001073946.1:p.Thr703Met	VAF 2.9% (23 reads)	

VAF across samples	AF	Fetal biopsy	P1	P2	P3	P4
4_182688238_C_T	51.67%	43.62%	51.70%	52.80%	52.56%	49.21%

Case 5

Chromosomal location	Clone	Sample	VAF	Gene	HGVSc	HGVSp	cfDNA analysis	Fetal Fraction
5_141389717_G_T	0	P2	7.14 %	PCDHGB4	NM_003736.4:c.1833G>T	NP_003727.1:p.Glu611Asp	no	
6_30705728_C_A	0	P2	7.29 %	MDC1,MDC1-AS1	NM_014641.3:c.3455G>T,NR_133647.1:n.127+2535C>A	NP_055456.2:p.Arg1152Met,	VAF 0.8%(3 reads)+0.9%(6 reads)	
6_134171163_G_A	2	P1	19.38 %	SGK1	NM_001143676.3:c.1183C>T	NP_001137148.1:p.Arg395Ter	no	5.05 %
7_100894103_G_T	4	P4	20.16 %	ACHE	NM_000665.5:c.130C>A	NP_000656.1:p.Arg44Ser	no	
11_5200416_C_A	6	P3	14.53 %	OR51V1	NM_001004760.3:c.267G>T	NP_001004760.3:p.Trp89Cys	VAF 0.7% (2 reads on H3377DSX5)	
19_10928884_T_C	6	P3	9.25 %	TIMM29	NM_138358.4:c.62T>C	NP_612367.1:p.Val21Ala	no	

Case 6 (no cfDNA data)

Chromosomal location	Clone	Sample	VAF	Gene	HGVSc	HGVSp	Chorionic villus sample (CVS)
8_143728594_C_T	1	P1	29.85 %	FAM83H	NM_198488.5:c.867G>A	NP_940890.4:p.Ala289=	VAF 2.1% in CVS (2 reads)
2_167258150_T_A	3	P3	39 %	XIRP2	NM_001199143.2:c.1703T>A	NP_001186072.1:p.Leu568Ter	no
19_14441323_C_T	3	P3	22.68 %	PKN1	NM_002741.5:c.202C>T	NP_002732.3:p.Arg68Cys	no
1_17655900_G_T	4	P2	15.39 %	ARHGEF10L	NM_018125.4:c.2503G>T	NP_060595.3:p.Ala835Ser	no
3_105552518_G_T	4	P2	10.79 %	ALCAM	NM_001627.4:c.1597G>T	NP_001618.2:p.Val533Phe	no
12_119145399_G_T	4	P2	13.01 %	SRRM4	NM_194286.4:c.790G>T	NP_919262.2:p.Ala264Ser	no
1_11847334_G_-	5	P3	6.33 %	NPPA,NPPA-AS1	NM_006172.4:c.229delC,NR_037806.1:n.1480-96delG	NP_006163.1:p.Leu77Serfs*35,	no
1_197356897_G_C	5	P3	5.1 %	CRB1	NM_201253.3:c.1055G>C	NP_957705.1:p.Gly352Ala	no
3_49662962_G_A	5	P3	5.0 %	BSN	NM_003458.4:c.10804G>A	NP_003449.2:p.Ala3602Thr	no
14_23534993_G_T	5	P3	8.42%	ZFHX2	NM_033400.3:c.333C>A	NP_207646.2:p.Ser111Arg	VAF 2.3% in CVS (2 reads)

Supplementary Figure 1. Variant allele frequency jitter plots illustrating postzygotic small sequence variant clones across different samples per case. AF = amniotic fluid, CVS = chorionic villus sampling, P1-P4 = placental biopsies from different quadrants, UC = umbilical cord blood.

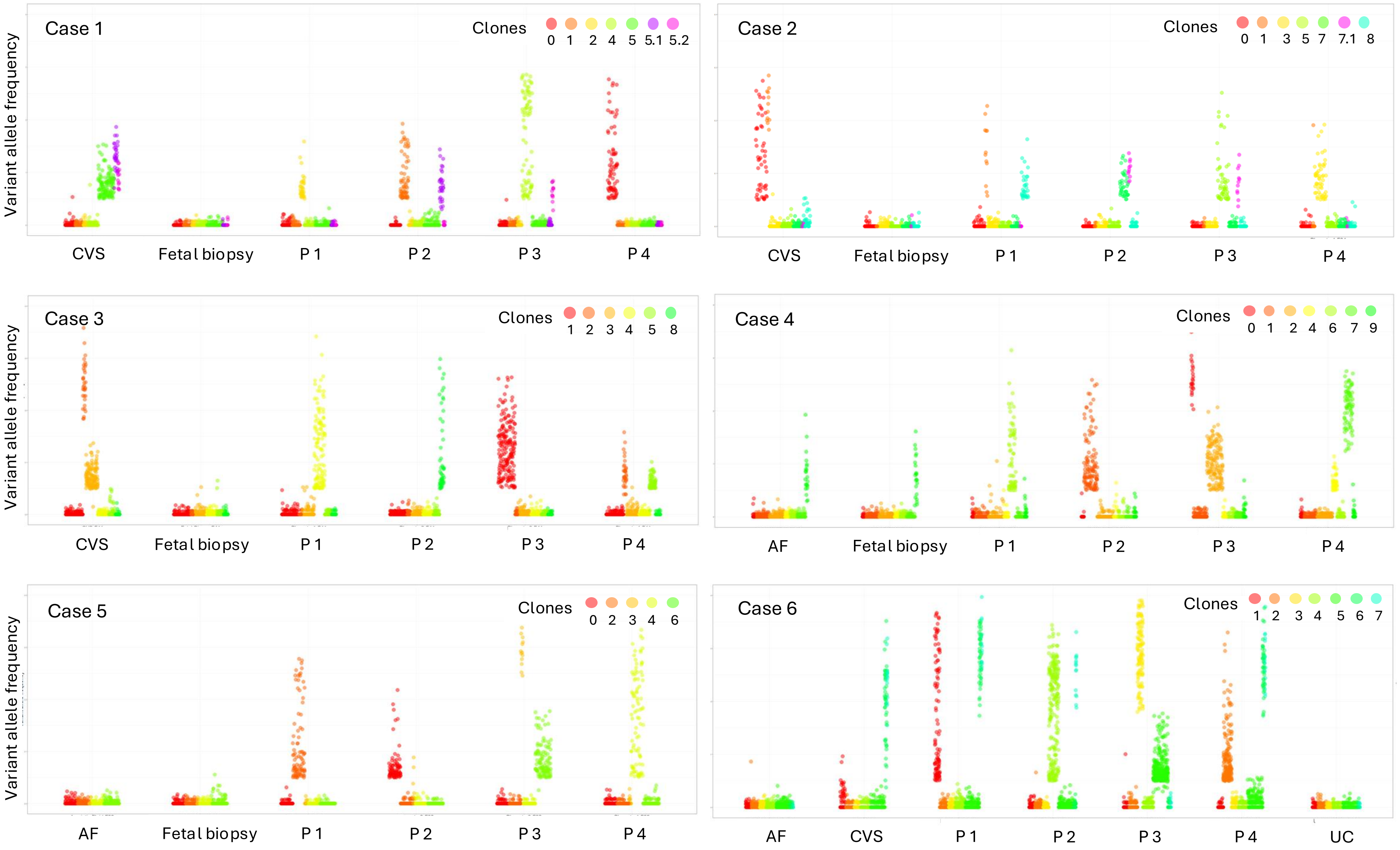

Supplementary Figure 2. Postzygotic copy number variants detected in placental biopsies. In case 3, duplications were observed at 1p13.2 (187 kb), 6q15 (173 kb), and 6p22.1 (103 kb). In case 6, Trisomy 21 and a duplication at 1q32.3 (292 kb) were identified. dup = duplication, kb = kilobase.

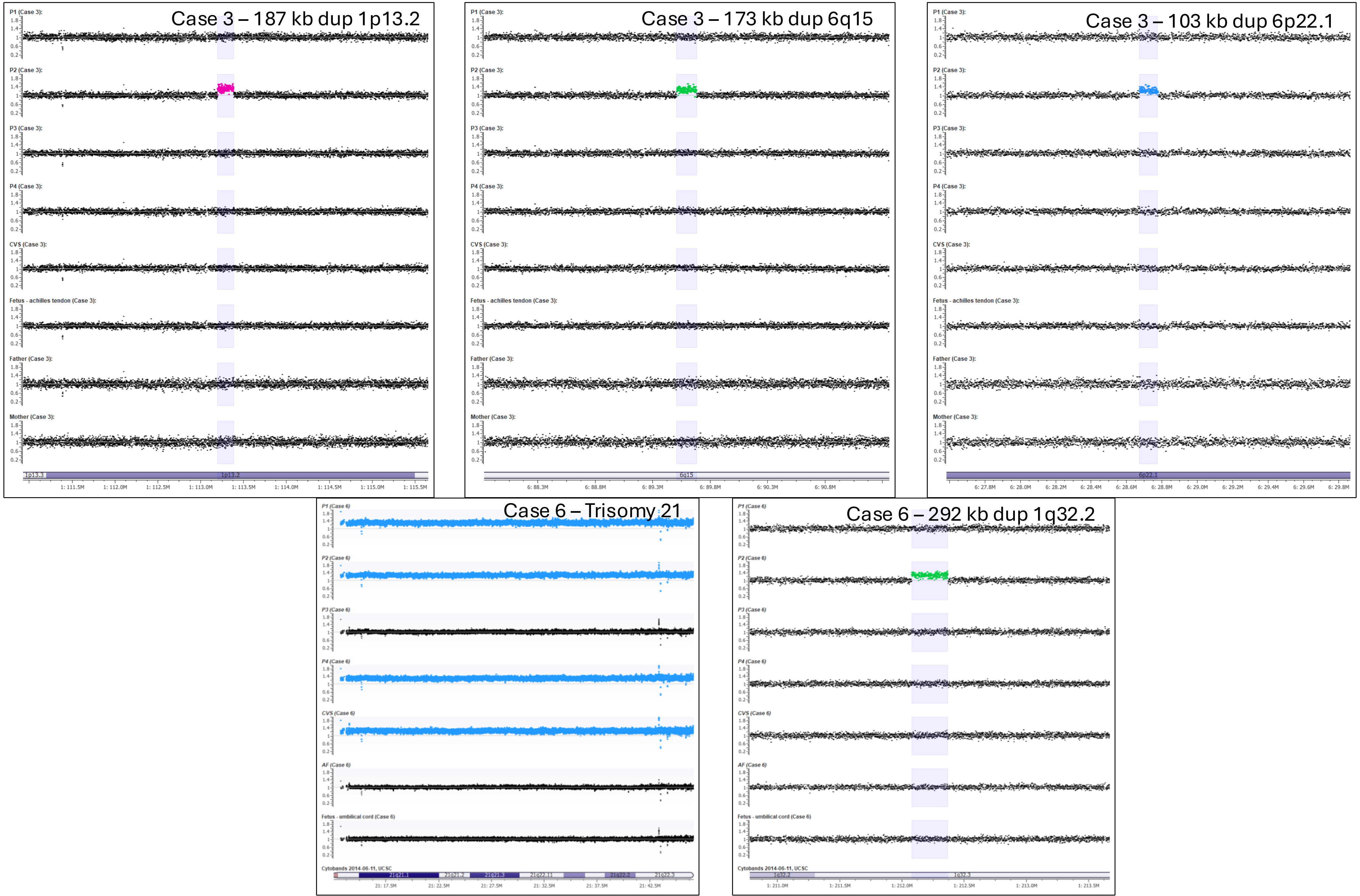

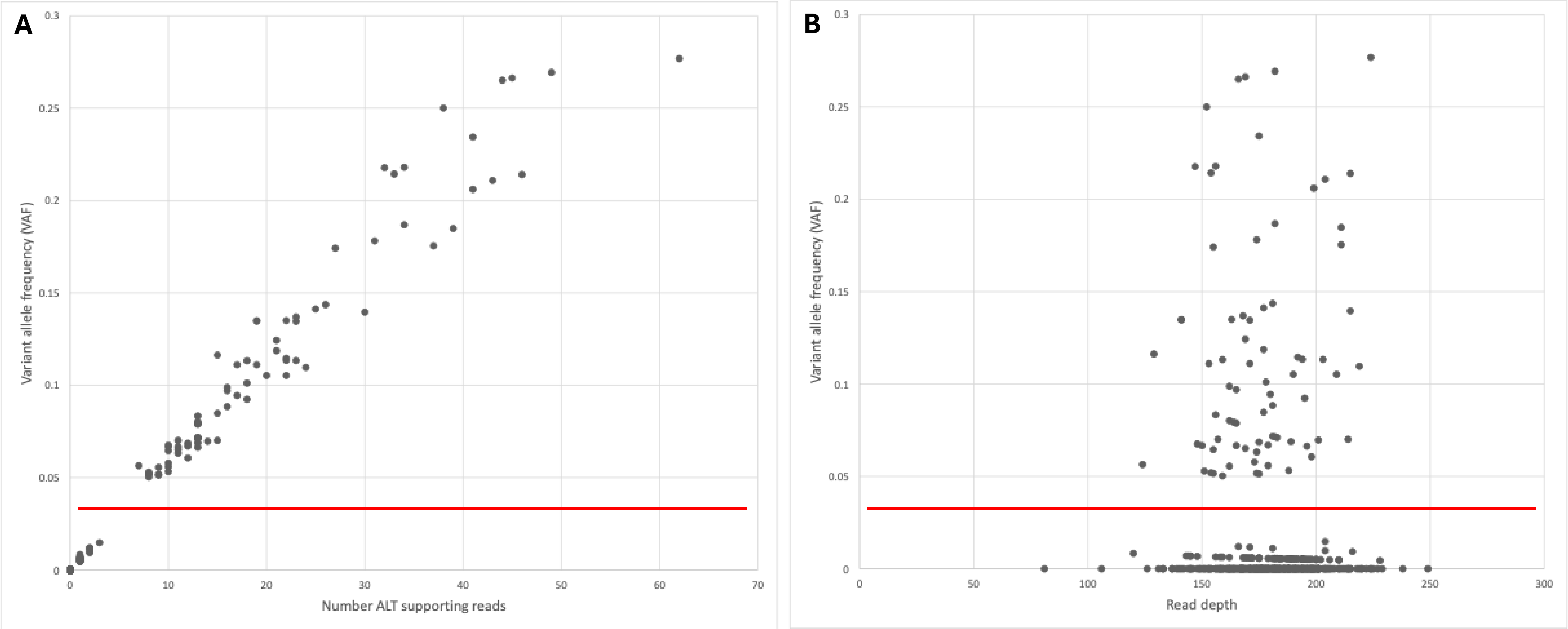

Supplementary Figure 3. Quality metrics utilized in establishing thresholds for variant filtering in this study. The association between variant read depth and variant allele frequency (VAF) (A), as well as VAF and alternative allele (ALT) supporting reads (B), demonstrates a clear separation between true somatic variants and the background sequencing artefacts (highlighted by the red line).

Supplementary Figure 4. Pile-up plots of selected postzygotic small sequence variants representing each clonal expansion identified in Case 1 across multiple tissue samples, including fetal biopsy, chorionic villus sampling (CVS), and placenta. Panels display read depth, coverage, and aligned sequencing reads to illustrate the presence and distribution of these variants across different tissues.

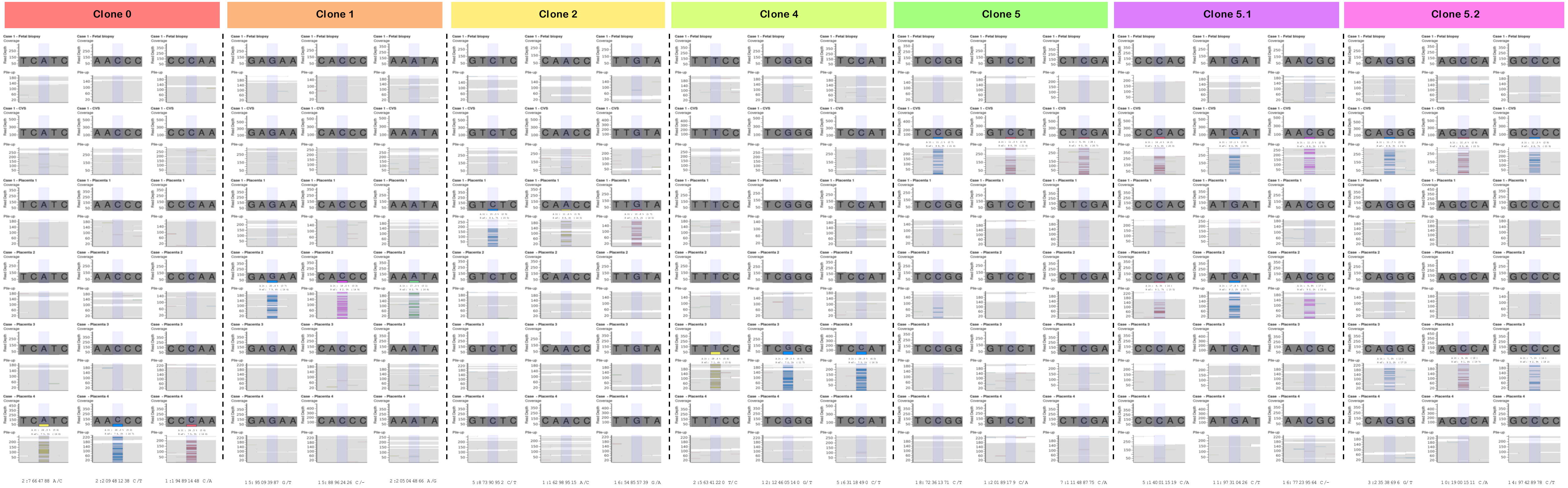

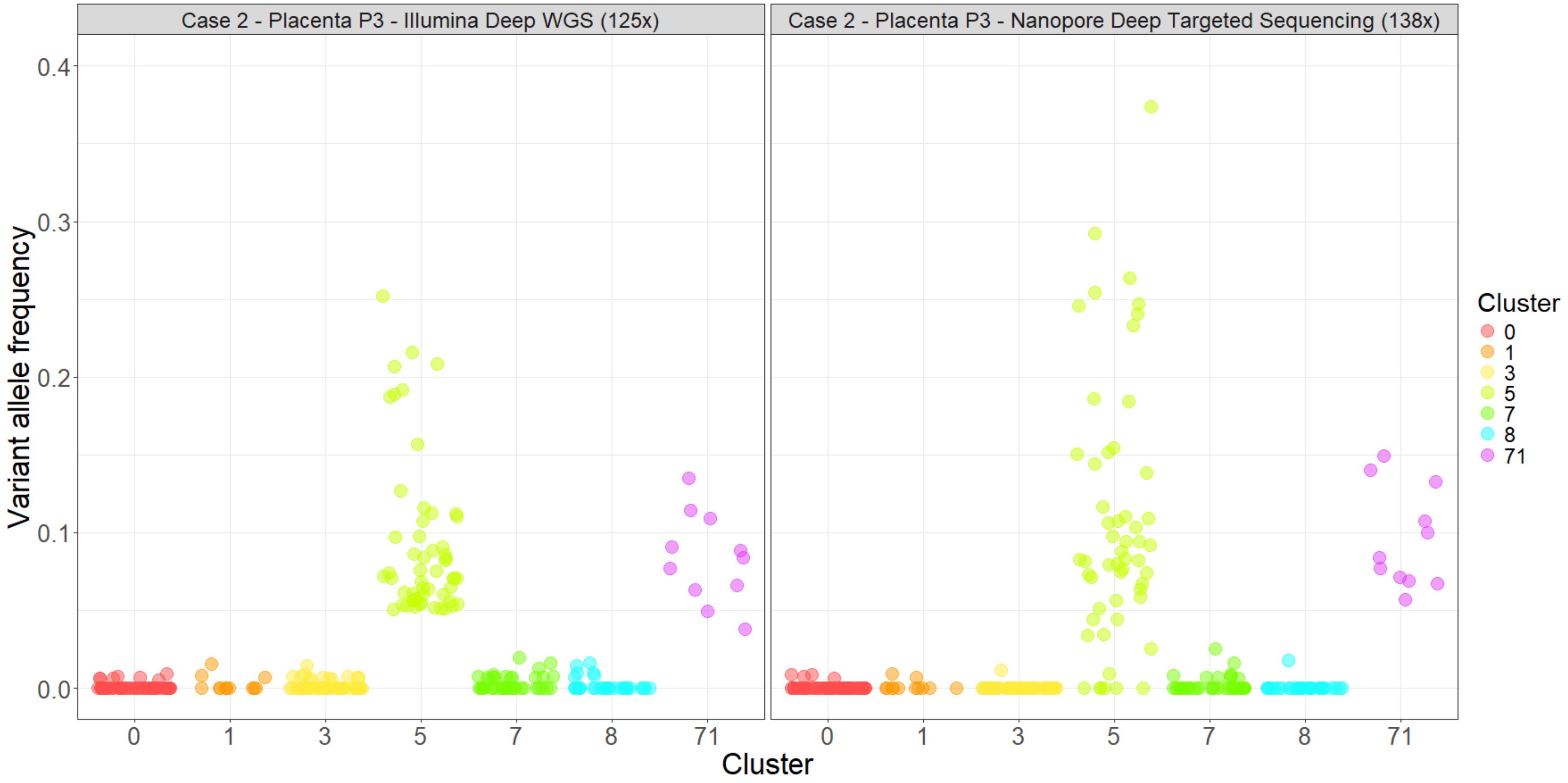

Supplementary Figure 5.

Verification of postzygotic variant clusters using targeted long-read sequencing. To confirm the presence of private placental clones identified by whole-genome sequencing, we performed deep targeted sequencing of the P3 biopsy from Case 2 using Oxford Nanopore Technology (ONT). Adaptive sampling was applied to selectively enrich for 254 genomic loci corresponding to postzygotic small sequence variants (PZVs) previously identified in the research dataset. The Nanopore sequencing confirmed the presence of the same somatic variant clusters, consistent with the clonal architecture observed in the Illumina-based WGS data. While minor differences in variant allele frequencies (VAFs) were observed, as expected due to differences in sequencing depth and technology, the overall variant patterns and clonal structure were concordant. This independent validation supports the robustness of the variant calling pipeline and the biological authenticity of the detected placental clones.

Supplementary Figure 6.

Chorionic villus samples (CVS) from Cases 1–3 were independently sequenced as part of routine clinical diagnostics at standard genome coverage, using a separate library preparation from that employed in the high-depth WGS.

Somatic variant clusters identified in the deep WGS dataset were consistently reproduced in the clinical sequencing data. Although variant allele frequencies (VAFs) showed greater variance in the lower-coverage clinical data, the same patterns were clearly detected. This reproducibility across independently processed datasets provides strong, orthogonal support for the presence of the identified clonal expansions in early placental tissue. It further reinforces the robustness of our somatic variant calling strategy and confirms that these findings are not artefacts.

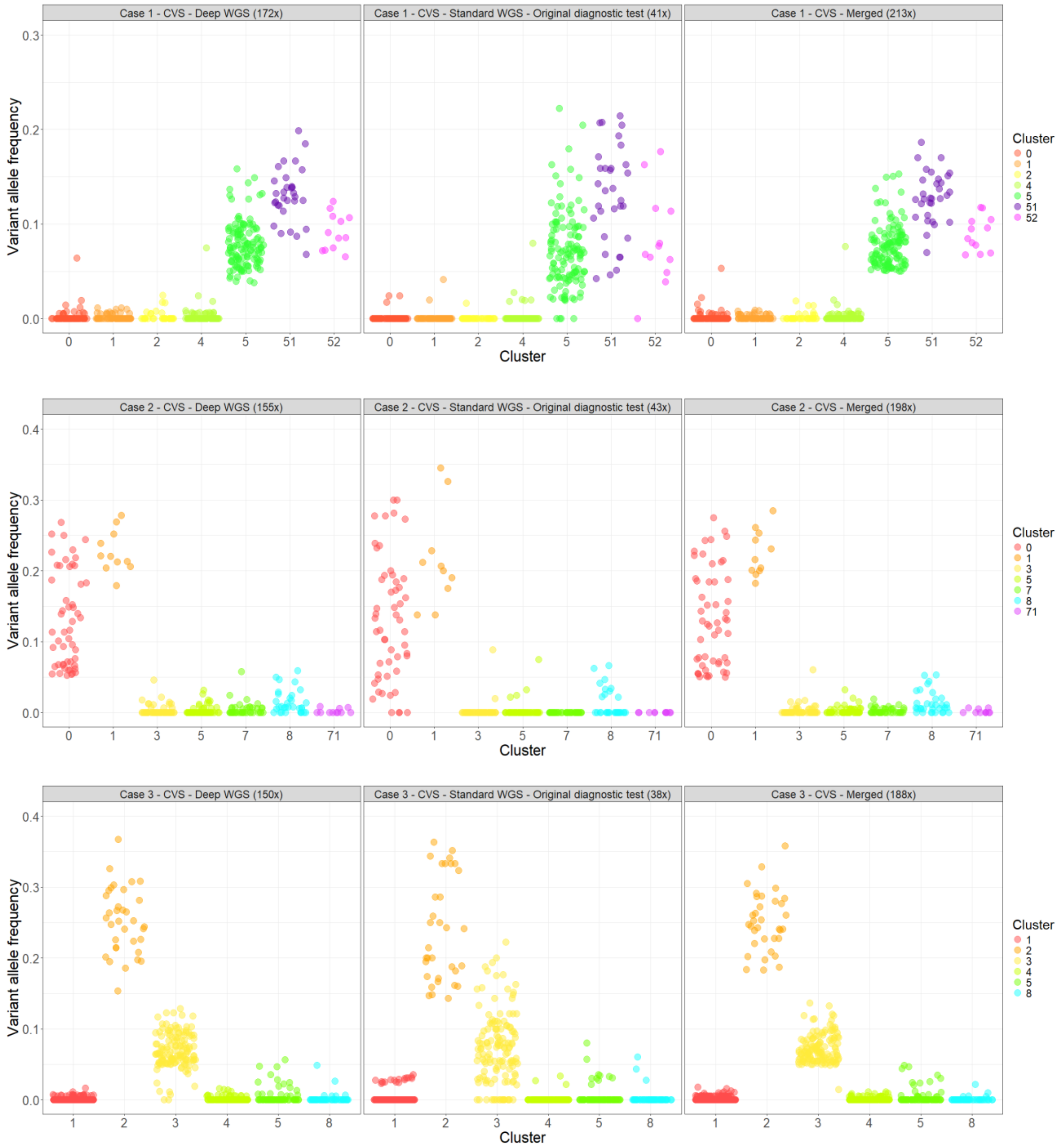

Supplement: Supplementary file 1 — Supplementary Information [file 41467_2025_63296_MOESM1_ESM.pdf]
