# Supplementary material for: Deep genome sequencing reveals extensive genetic heterogeneity in early human placentas
Source: Nat Commun. 2025 Aug 23;16:7873. doi: 10.1038/s41467-025-63296-3 (PMC12374992; doi:10.1038/s41467-025-63296-3)
Supplement: Supplementary file 2 — Reporting Summary [file 41467_2025_63296_MOESM2_ESM.pdf]

nature portfolio

Corresponding author(s): Ieva Miceikaite, Martin Jakob Larsen

Last updated by author(s): Jun 27, 2025

Reporting Summary

Nature Portfolio wishes to improve the reproducibility of the work that we publish. This form provides structure for consistency and transparency in reporting. For further information on Nature Portfolio policies, see our Editorial Policies and the Editorial Policy Checklist.

Statistics

For all statistical analyses, confirm that the following items are present in the figure legend, table legend, main text, or Methods section.

n/a	Confirmed	
☐	☒	The exact sample size (*n*) for each experimental group/condition, given as a discrete number and unit of measurement
☐	☒	A statement on whether measurements were taken from distinct samples or whether the same sample was measured repeatedly
☒	☐	The statistical test(s) used AND whether they are one- or two-sided<br>*Only common tests should be described solely by name; describe more complex techniques in the Methods section.*
☒	☐	A description of all covariates tested
☐	☒	A description of any assumptions or corrections, such as tests of normality and adjustment for multiple comparisons
☐	☒	A full description of the statistical parameters including central tendency (e.g. means) or other basic estimates (e.g. regression coefficient) AND variation (e.g. standard deviation) or associated estimates of uncertainty (e.g. confidence intervals)
☒	☐	For null hypothesis testing, the test statistic (e.g. *F*, *t*, *r*) with confidence intervals, effect sizes, degrees of freedom and *P* value noted<br>*Give P values as exact values whenever suitable.*
☒	☐	For Bayesian analysis, information on the choice of priors and Markov chain Monte Carlo settings
☒	☐	For hierarchical and complex designs, identification of the appropriate level for tests and full reporting of outcomes
☐	☒	Estimates of effect sizes (e.g. Cohen's *d*, Pearson's *r*), indicating how they were calculated

Our web collection on statistics for biologists contains articles on many of the points above.

Software and code

Policy information about availability of computer code

Data collection	No software for data collection was used; No custom code was developed in this study.
Data analysis	Used software information: Illumina DRAGEN Bio-IT Platform v4.03; bedtools version 2.31.1; bcftools version 1.22; VarSeq v2.3.0 (Golden Helix); VafVaf v0.4 (https://mjlarsen.shinyapps.io/VafVaf-v0-4); PyClone 0.13.1 (https://github.com/Roth-Lab/pyclone); COSMIC SigProfiler (https://cancer.sanger.ac.uk/signatures); Dorado v0.9.6

For manuscripts utilizing custom algorithms or software that are central to the research but not yet described in published literature, software must be made available to editors and reviewers. We strongly encourage code deposition in a community repository (e.g. GitHub). See the Nature Portfolio guidelines for submitting code & software for further information.

Data

Policy information about availability of data

All manuscripts must include a data availability statement. This statement should provide the following information, where applicable:

- Accession codes, unique identifiers, or web links for publicly available datasets
- A description of any restrictions on data availability
- For clinical datasets or third party data, please ensure that the statement adheres to our policy

The processed somatic variant calls generated from genome sequencing of the study samples are publicly available at the Figshare repository: https://doi.org/10.6084/m9.figshare.27281799.v1. The raw sequencing data generated during this study are not available due to ethical and legal restrictions under the

General Data Protection Regulation (GDPR) and Danish national legislation on the protection of personal data. These data contain identifiable genetic information and cannot be shared publicly or upon request, including under confidentiality agreements. Source data are provided with this paper.

Research involving human participants, their data, or biological material

Policy information about studies with human participants or human data. See also policy information about sex, gender (identity/presentation), and sexual orientation and race, ethnicity and racism.

Reporting on sex and gender	This study focused on human placental tissues from pregnant individuals. The sex of the fetus was not a specific variable of investigation, and placentas from both male and female fetuses were included. Our study primarily examined genetic variations in placental tissues, with no direct assessment of the effect of sex. Biological sex of each fetus (placenta) is depicted in the Supplementary Table1 as XX, XY or X0, as it was determined from genetic sequencing data. The sex of pregnant individuals was assigned based on medical records as female; no additional gender identity data were collected. The study did not involve sex- or gender-specific research questions or stratified analyses, as the primary aim was to investigate placental mosaicism and postzygotic variant distribution in a limited sample cohort.
Reporting on race, ethnicity, or other socially relevant groupings	Race or ethnicity were not variables assessed in this study, as the analysis of genetic heterogeneity in placental tissues is equally relevant for all pregnancies, despite race, ethnicity or other social groupings.
Population characteristics	This study involved six pregnant women, ages 25-33 years, who had undergone first- or second-trimester chorionic villus sampling or second-trimester amniocentesis, and their partners. All included cases had indications for prenatal genetic testing and causative chromosomal or sequence variants were identified for most of them. The gestational ages at the time of sample collection varied, and are provided for all included samples in Table 1.
Recruitment	This study involved analysis of biological samples from six pregnancies recruited at the Department of Clinical Genetics and the Department of Obstetrics and Gynecology, Odense University Hospital, Denmark. All cases included in the study were pregnancies that underwent invasive diagnostic testing due to abnormal ultrasound findings or high-risk screening results. Before enrolment, all participants were informed by qualified personnel about the genetic analysis being performed on the placental tissue and the confidentiality of their personal information, and they signed informed consent forms.<br>As a result of the clinical referral-based recruitment, there is an inherent ascertainment bias toward pregnancies with abnormal findings. This and the small cohort size (n = 6), even though unlikely in this particular study, might limit the generalizability of the findings to the broader population of healthy pregnancies. These biases were considered in the interpretation of results, and the study's conclusions are framed accordingly.
Ethics oversight	The project was approved by the Region of Southern Denmark Research Ethics Committee (Project-ID: S-20190027), and complied with the General Data Protection Regulation (GDPR).

Note that full information on the approval of the study protocol must also be provided in the manuscript.

Field-specific reporting

Please select the one below that is the best fit for your research. If you are not sure, read the appropriate sections before making your selection.

☒ Life sciences ☐ Behavioural & social sciences ☐ Ecological, evolutionary & environmental sciences

For a reference copy of the document with all sections, see nature.com/documents/nr-reporting-summary-flat.pdf

Life sciences study design

All studies must disclose on these points even when the disclosure is negative.

Sample size	The study included six pregnancies, each with 4 postgestational placental biopsies, 1 amniotic fluid and/or CVS sample, 1 maternal plasma sample (if available), and parental blood samples (when available). No formal sample size calculation was performed. Cases were selected based on availability of multi-sample access and clinical indication. Despite the small number, the dataset enabled high-resolution spatial and temporal analysis of placental mosaicism. The consistent patterns observed across gestation support the robustness and relevance of the findings.
Data exclusions	No sequencing genetic data were excluded from the analysis. Variant filtering were carried out as described in the method section.
Replication	Key findings were confirmed across independent sample types (e.g., CVS, amniotic fluid, maternal plasma, fetal tissue) within each case. In three cases, we performed whole-genome sequencing on separately prepared diagnostic and research libraries from the same samples, consistently detecting the same somatic variant clusters. While no formal replication in an external cohort was performed, the reproducibility across multiple tissue types, timepoints, and sequencing preparations supports the robustness of the results. All attempts at internal replication were successful.
Randomization	Not relevant, as we are not investigating group effects
Blinding	Blinding was not relevant in the current study, as this was a genomic study with no intervention groups or comparative treatments. All samples were analyzed using standardized pipelines, and data interpretation was based on objective bioinformatic variant calling and sequencing results. Clinical data were incorporated post hoc to contextualize genetic findings but did not influence variant detection or initial

analysis.

Reporting for specific materials, systems and methods

We require information from authors about some types of materials, experimental systems and methods used in many studies. Here, indicate whether each material, system or method listed is relevant to your study. If you are not sure if a list item applies to your research, read the appropriate section before selecting a response.

Materials & experimental systems

n/a	Involved in the study
☒	☐ Antibodies
☒	☐ Eukaryotic cell lines
☒	☐ Palaeontology and archaeology
☒	☐ Animals and other organisms
☒	☐ Clinical data
☒	☐ Dual use research of concern
☒	☐ Plants

Methods

n/a	Involved in the study
☒	☐ ChIP-seq
☒	☐ Flow cytometry
☒	☐ MRI-based neuroimaging

Plants

Seed stocks	*Report on the source of all seed stocks or other plant material used. If applicable, state the seed stock centre and catalogue number. If plant specimens were collected from the field, describe the collection location, date and sampling procedures.*
Novel plant genotypes	*Describe the methods by which all novel plant genotypes were produced. This includes those generated by transgenic approaches, gene editing, chemical/radiation-based mutagenesis and hybridization. For transgenic lines, describe the transformation method, the number of independent lines analyzed and the generation upon which experiments were performed. For gene-edited lines, describe the editor used, the endogenous sequence targeted for editing, the targeting guide RNA sequence (if applicable) and how the editor was applied.*
Authentication	*Describe any authentication procedures for each seed stock used or novel genotype generated. Describe any experiments used to assess the effect of a mutation and, where applicable, how potential secondary effects (e.g. second site T-DNA insertions, mosiacism, off-target gene editing) were examined.*
